# Probability of Response as Defined by a Clinical Decision Support Tool Is Associated With Lower Healthcare Resource Utilization in Vedolizumab-Treated Patients With Crohn’s Disease

**DOI:** 10.1093/crocol/otac048

**Published:** 2022-12-03

**Authors:** Parambir S Dulai, Yaping Wan, Zhongwen Huang, Michelle Luo

**Affiliations:** Division of Gastroenterology and Hepatology, Feinberg School of Medicine Northwestern University, Chicago, Illinois, USA; Takeda Pharmaceuticals U.S.A., Deerfield, Illinois, USA; Takeda Pharmaceuticals U.S.A., Deerfield, Illinois, USA; Takeda Pharmaceuticals U.S.A., Deerfield, Illinois, USA

**Keywords:** clinical decision support tool, Crohn’s disease, healthcare resource utilization, vedolizumab

## Abstract

**Background:**

A previously developed clinical decision support tool (CDST) identified patients with Crohn’s disease (CD) most likely to respond to vedolizumab. This study evaluated the ability of the CDST to predict real-world healthcare resource utilization (HRU).

**Methods:**

The Optum and Truven healthcare databases were searched for patients with CD treated with vedolizumab (Optum, *n* = 358; Truven, *n* = 1445) or an anti-tumor necrosis factor (TNF) agent (Optum, *n* = 814). Patients were stratified using the 5-variable (prior bowel surgery, prior fistulizing disease, prior anti-TNF exposure, albumin, C-reactive protein) and a new modified 3-variable (without laboratory data) CDST. Annualized expenditures and HRU were compared with both CDSTs across response probability groups for a 12-month period.

**Results:**

In the Optum data set, the 5- and 3-variable CDSTs identified lower rates of surgery or hospitalization in CD patients with higher probability of vedolizumab response. Per-patient total costs were 2.5 times lower for CD patients with high versus low probability of vedolizumab response ($12 943 vs $32 931). The 5- and 3-variable CDSTs did not consistently identify anti-TNF-treated CD patients with higher HRU. The 3-variable CDST also identified vedolizumab-treated CD patients with higher probability of response and lower probability for surgery or hospitalization in the Truven data set.

**Conclusions:**

The 5-variable CDST identified CD patients treated with vedolizumab, but not an anti-TNF agent, at higher risk for HRU. The 3-variable CDST offers similar performance but more flexibility by removing laboratory data requirements for prediction. These validated CDSTs can be integrated into population health monitoring algorithms using real-world data.

## Graphical Abstract

**Graphical Abstract:**
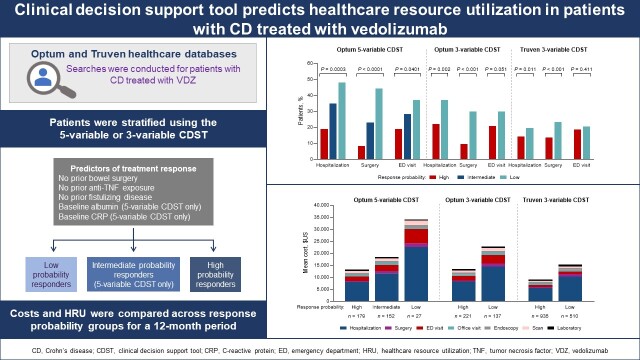

## Introduction

Crohn’s disease (CD) is a chronic and relapsing inflammatory bowel disease (IBD) characterized by abdominal pain, diarrhea, weight loss, and fatigue.^1,2^ CD can be associated with high morbidity and substantial cost burden,^3^ primarily driven by the development of disease-related complications and need for hospitalization and/or surgery.^2,4,5^ Approximately 70% of patients with CD require at least 1 surgical intervention, which accounts for the majority of hospitalizations.^4^ Of the total annual costs for CD, nearly 40% are attributable to surgery^4^ and 31% to hospitalization.^5^ Therefore, primary therapeutic goals in CD are to improve symptomatic activity and patient well-being while reducing the risk of disease progression and associated complications.^1,6^ These improvements will likely also lower healthcare resource utilization (HRU) and disease-related costs.

Vedolizumab is a gut-selective, humanized monoclonal antibody that specifically binds to the α_4_β_7_ integrin and is approved for the treatment of adult patients with moderately to severely active ulcerative colitis or CD.^7,8^ Vedolizumab efficacy in achieving clinical remission has been demonstrated in phase 3 clinical trials^9–12^ and its real-world effectiveness has been confirmed in multiple observational cohort studies.^13,14^ Nonetheless, some patients treated with vedolizumab may still experience disease progression and complications that result in hospitalization and/or surgery. Early identification of potential at-risk patients could help direct them to treatment strategies that are proven to reduce the risk of complications, such as treat-to-target monitoring.

A clinical decision support tool (CDST) has been developed using data from the vedolizumab GEMINI 2 phase 3 trial and validated using data from the real-world evidence VICTORY consortium to identify CD patients who are most likely to respond to vedolizumab therapy in routine clinical practice.^15^ Preliminary data suggest that this tool is also able to predict the risk of CD-related surgery.^16^ Therefore, this CDST has the potential to identify CD patients who remain at risk for disease-related complications while on vedolizumab and who might benefit the most from evolving treatment strategies. However, before broad implementation of this tool, further validation of its ability to predict disease-related complications and healthcare expenditure is needed in the real-world setting. Furthermore, there is a need for real-world assessment of whether this CDST is drug specific or more broadly applicable to other biologic CD treatments such as anti-tumor necrosis factor (TNF) therapy.

Here, we evaluated the ability of this CDST to predict differences in CD-related hospitalization, surgery, and other HRU in CD patients treated with vedolizumab, and further assessed whether this vedolizumab CDST-predicted outcomes for CD patients treated with an anti-TNF agent, using data from 2 real-world HRU data sets.

## Methods

### Data Set

CD patients treated with vedolizumab or an anti-TNF agent were identified from the Optum database for initial CDST real-world HRU data validation and comparison. Recognizing that the laboratory variables included in the 5-variable CDST may not be routinely available when applying the CDST in clinical practice, a modified 3-variable CDST that does not require laboratory values was assessed in CD patients treated with vedolizumab in the Optum database and then further validated against similar patients in the Truven MarketScan database.

Optum I3 Clinformatics Data Mart is an administrative health claims database for members of a large US national managed care company affiliated with Optum. This database includes approximately 15–18 million annual covered lives for a total of roughly 57 million covered lives over a 10-year period (from January 2007 to December 2017). The Truven Health Analytics Database, a part of the IBM MarketScan Databases, is a US nationally representative claims and Medicare supplemental database that consists of medical and pharmacy claims of >150 employers, including 100 health plans, representing approximately 170 million covered lives.

### Study Population

Eligible patients were adults who were ≥18 years old on their vedolizumab or anti-TNF agent initiation date and were treated with vedolizumab or an anti-TNF agent between May 1, 2014 and March 31, 2018. Biologic-naive patients and patients that had received prior anti-TNF were included and were not divided by prior anti-TNF exposure. Patients had 2 separate diagnoses of CD 30 days apart after January 1, 2000 and before their vedolizumab or anti-TNF initiation date, and had ≥6 months of pre- and post-index continuous enrollment. The specific codes used to identify the cohorts from both the Optum and Truven data sets are provided in Supplementary Tables S1–S8.

### Clinical Decision Support Tool

**Figure 1.**
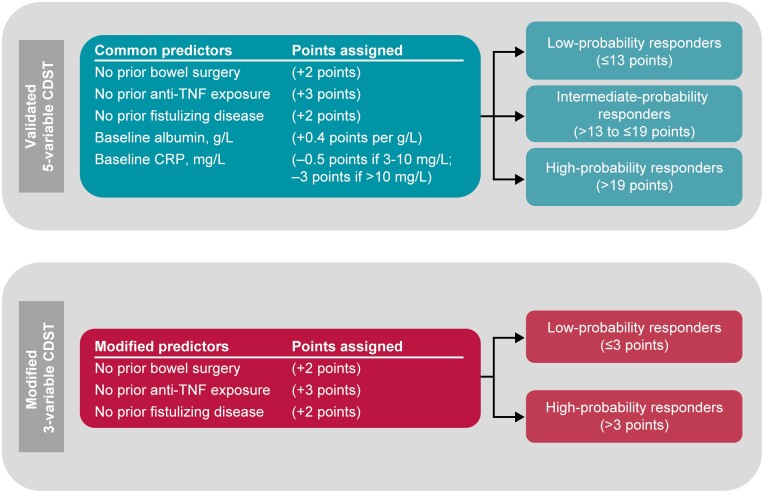
Full 5-variable and modified 3-variable vedolizumab CDSTs for CD. Abbreviations: CD, Crohn’s disease; CDST, clinical decision support tool; CRP, C-reactive protein; TNF, tumor necrosis factor.

The 5-variable CDST^17^ was applied to the Optum data set with available laboratory data (Figure 1). Patient CDST scores were calculated using baseline laboratory data obtained prior to treatment initiation and included 3 clinical variables (no prior bowel surgery [+2 points], no prior fistulizing disease [+2 points], no prior anti-TNF exposure [+3 points]) and 2 laboratory values (albumin [+0.4 point per g L^−1^], C-reactive protein [CRP; −0.5 points if CRP was 3.0–10.0 mg L^−1^, −3 points if CRP was >10.0 mg L^−1^]). Patients were then stratified into high probability of response (defined as total points >19), intermediate probability of response (defined as total points >13 to ≤19), and low probability of response (defined as total points ≤13). This stratification was performed for patients treated with vedolizumab or an anti-TNF agent to assess whether the CDST-predicted associations between probability of response and HRU were drug specific.

A modified CDST (Figure 1) based only on the 3 clinical variables included in the 5-variable CDST was created in recognition of the fact that laboratory data may not be routinely available in all claims or electronic health record data sets. For the 3-variable CDST assessments, high and low probabilities of response were defined as total points >3 and total points ≤3, respectively. The cutoff of 3 points to separate high- and low-probability responders was derived from a post hoc assessment of the GEMINI 2 data set, which showed a fair correlation with the full 5-variable CDST for both high- and low-probability responders (Supplementary Table S9). Since the 3-variable CDST had reduced data input relative to the 5-variable CDST due to lack of laboratory data, a model with 2 vedolizumab response groups (low/high) was considered most feasible.

### Outcomes

Event proportions and event incidence rates of CD-related hospitalization, surgery, and emergency department (ED) visits and annualized per-patient costs of CD-related hospitalization, surgery, and total healthcare expenditures within the first 12 months of vedolizumab or anti-TNF agent initiation were compared across response probability groups. These outcomes were identified in the Optum and Truven databases using prespecified coding criteria.

Patients who had any hospitalization claim with a CD-related diagnosis (Supplementary Table S2) and who were under observation (Supplementary Table S7) during the 12 months after treatment initiation were categorized as having a hospitalization. Patients with CD-related surgeries were identified using Current Procedural Terminology (CPT) codes (Supplementary Table S8), and patients with a medical claim for a revenue code (0450–0459, 0981) indicating ED as the place of service were categorized as having an ED visit. Any ED visit that resulted in hospitalization or surgery was counted as hospitalization or surgery, respectively.

Total healthcare expenditures included the sum of expenditures for hospitalization, surgery, ED visits, office visits, endoscopies, scans, and laboratory tests. Office visits were identified using CPT codes 99211–99215; endoscopies and scans were identified during the 12 months after treatment initiation by the CPT codes listed in Supplementary Tables S10 and S11; and laboratory tests were identified during follow-up by claims with revenue center codes 0300–0319.

The expenditures of each claim were converted to 2017 US dollars ($) using the conversion factors constructed from the average of Consumer Price Index of 12 months for 2015 and 2016, then annualized according to the following rules: actual expenditure in 2017 $ after the treatment initiation date if the interval between the treatment initiation date and the end of continuous insurance eligibility was >12 months; or annualized expenditure in 2017 $ computed as (expenditure in 2017 $)/(end date of insurance eligibility − treatment initiation data + 1) × 365.25 if the interval between the treatment initiation date and the end of continuous insurance eligibility was <12 months.

### Statistical Analysis

CD patients treated with vedolizumab or an anti-TNF agent in the Optum data set were classified and compared using the full 5-variable and modified 3-variable CDSTs. Patients treated with vedolizumab in the Truven data set were also classified and analyzed using the modified 3-variable CDST to compare for consistency in outcomes in the Optum data set.

Statistical analyses were performed using SAS software. Categorical or binary variables (ie, CD-related events) are reported as proportions or percentages, whereas continuous variables (ie, annualized costs) are reported as mean values (± SD). *P* values were calculated using Pearson’s χ^2^-test for categorical variables, and either 1-way analysis of variance among 3 responder groups or 2-sample *t*-tests between 2 responder groups for continuous variables.

### Ethical Considerations

This study was a retrospective analysis of anonymized administrative claims data from 2 large US healthcare claims databases (Truven and Optum). No specific ethical approval for use of these data was required.

## Results

### Baseline and Demographic Characteristics

A total of 358 and 1445 CD patients treated with vedolizumab from the Optum and Truven databases, respectively, were included in the study (Table 1). Patients were stratified into high (*n* = 179), intermediate (*n* = 152), and low (*n* = 27) probabilities of response groups using the Optum data set based on the full CDST, and into high (*n* = 221) and low (*n* = 137) probabilities of response groups using the Optum data set based on the modified CDST. Patients were stratified into high (*n* = 935) and low (*n* = 510) probabilities of response groups using the Truven database based on the modified CDST.

**Table 1.** Baseline demographics and characteristics of patients with CD treated with vedolizumab (Optum and Truven data sets).

	Optum data set							Truven data set		
	5-variable CDST				3-variable CDST			3-variable CDST		
	Probability of response			*P* value	Probability of response		*P* value	Probability of response		*P* value
	High (*n* = 179)	Int (*n* = 152)	Low (*n* = 27)		High (*n* = 221)	Low (*n* = 137)		High (*n* = 935)	Low (*n* = 510)	
Female, *n* (%)	97 (54.2)	96 (63.2)	20 (74.1)	.070	125 (56.6)	88 (64.2)	.151	532 (56.9)	295 (57.8)	.729
Age in years, mean (SD)	46.3 (16.8)	45.2 (14.9)	36.9 (13.6)	.016^*^	46.6 (17.0)	42.8 (13.9)	.023^*^	43.8 (14.7)	42.8 (13.7)	.232
Disease duration in years, mean (SD)	3.6 (3.7)	4.9 (4.1)	5.5 (4.7)	.004^*^	3.4 (3.4)	5.8 (4.5)	<.001^*^	3.2 (3.0)	5.0 (3.7)	<.001^*^
CD-related hospitalization,^a^*n* (%)	41 (22.9)	52 (34.2)	19 (70.4)	<.001^*^	49 (22.2)	63 (46.0)	<.001^*^	129 (13.8)	147 (28.8)	<.001^*^
CD-related surgery,^a^*n* (%)	8 (4.5)	29 (19.1)	14 (51.9)	<.001^*^	5 (2.3)	46 (33.6)	<.001^*^	34 (3.6)	183 (35.9)	<.001^*^
Fistula,^a^*n* (%)	10 (5.6)	40 (26.3)	16 (59.3)	<.001^*^	3 (1.4)	63 (46.0)	<.001^*^	5 (0.5)	54 (10.6)	<.001^*^
Stricture,^a^*n* (%)	0 (0.0)	2 (1.3)	0 (0.0)	.256	1 (0.5)	1 (0.7)	.732	1 (0.1)	6 (1.2)	.005^*^
History of fistulizing disease, *n* (%)	15 (8.4)	59 (38.8)	21 (77.8)	<.001^*^	4 (1.8)	91 (66.4)	<.001^*^	36 (3.9)	323 (63.3)	<.001^*^
IMM- or anti-TNF naive, *n* (%)	49 (27.4)	10 (6.6)	0 (0.0)	<.001^*^	53 (24.0)	6 (4.4)	<.001^*^	254 (27.2)	12 (2.4)	<.001^*^
Number of anti-TNF treatments received, *n* (%)										
0	72 (40.2)	14 (9.2)	0 (0.0)	<.001^*^	77 (34.8)	9 (6.6)	<.001^*^	376 (40.2)	24 (4.7)	<.001^*^
1	70 (39.1)	80 (52.6)	12 (44.4)		98 (44.3)	64 (46.7)		403 (43.1)	303 (59.4)	
≥2	37 (20.7)	58 (38.2)	15 (55.6)		46 (20.8)	64 (46.7)		156 (16.7)	183 (35.9)	
Concomitant IMM treatment, *n* (%)	48 (26.8)	37 (24.3)	11 (40.7)	.208	59 (26.7)	37 (27.0)	.949	241 (25.8)	154 (30.2)	.072
Concomitant CS treatment, *n* (%)	88 (49.2)	92 (60.5)	17 (63.0)	.081	117 (52.9)	80 (58.4)	.313	484 (51.8)	269 (52.7)	.721

Abbreviations: CD, Crohn’s disease; CDST, clinical decision support tool; CS, corticosteroid; IMM, immunomodulator; Int, intermediate; TNF, tumor necrosis factor.

^a^During the year before vedolizumab initiation.

^*^ *P* values are statistically significant.

Vedolizumab-treated patients classified as high-probability responders were older among Optum patients, but not among Truven patients; had shorter disease duration; were less likely to have had CD-related hospitalization, surgery, or fistula during the year before vedolizumab initiation; were less likely to have a history of fistulizing disease; and were more likely to be naive for treatment with immunomodulators. Significant differences in baseline characteristics were observed between high- and low-probability vedolizumab response groups (Table 1).

A total of 814 CD patients treated with an anti-TNF from the Optum database were included in the study (Supplementary Table S12). Patients were stratified into high (*n* = 650), intermediate (*n* = 158), and low (*n* = 6) probabilities of response groups using the Optum data set based on the full CDST, and into high (*n* = 726) and low (*n* = 88) probabilities of response groups using the Optum data set based on the modified CDST. Owing to the small number of low probability patients (*n* = 6) when using the 5-variable CDST, these patients were not included in the analyses.

### Healthcare Resource Utilization

#### Optum data set

**Figure 2.**
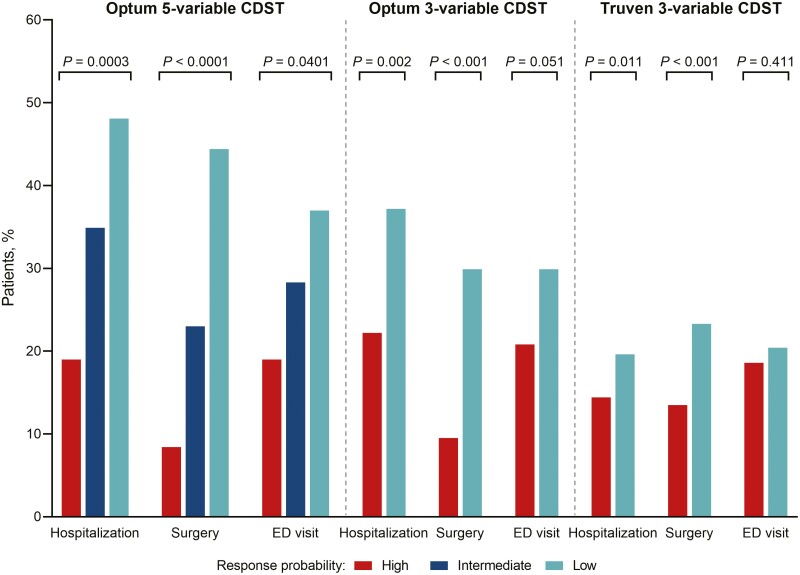
Healthcare resource utilization in patients with CD treated with vedolizumab (Optum and Truven data sets). Abbreviations: CD, Crohn’s disease; CDST, clinical decision support tool; ED, emergency department. *P* values are from χ^2^-tests; *P* < .05 is statistically significant.

When stratified by baseline probability of response, a significant association was observed when using the 5-variable CDST between predicted probability of response and HRU for vedolizumab-treated CD patients across all outcomes (hospitalization, surgery, ED visits). This pattern was also observed for the 3-variable CDST in the Optum data set (Figure 2), although for ED visits, the modified CDST did not reach statistical significance (*P* = .051). Rates of hospitalization and surgery were 2–4 times lower for vedolizumab-treated patients who had a high baseline probability of response compared with those with a low baseline probability of response using both the full CDST (hospitalization: 19.0% vs 48.1%, *P* < .001; surgery: 8.4% vs 44.4%, *P* < .001) and the modified CDST (hospitalization: 22.2% vs 37.2%, *P* = .002; surgery: 9.5% vs 29.9%, *P* < .001). Among anti-TNF-treated CD patients, the 5-variable CDST found a significant association between baseline probability of response and surgery or hospitalization but not ED visits; however, the strength of association was less than observed for the 5-variable CDST and vedolizumab-treated patients. For the 3-variable CDST, no association between anti-TNF responsive patients and hospitalization or ED visits was observed, demonstrating inconsistency in prediction in this patient cohort (Supplementary Figure S1).

#### Truven data set

HRU for patients treated with vedolizumab in the Truven database demonstrated a similar pattern to that observed with the Optum database patients categorized with the modified 3-variable CDST (Figure 2). A significantly lower proportion of the high probability of response group experienced a CD-related hospitalization (14.4% vs 19.6%, *P* = .011) or CD-related surgery (13.5% vs 23.3%, *P* < .001) during the 12 months after vedolizumab initiation.

### Healthcare Costs

#### Optum data set

**Figure 3.**
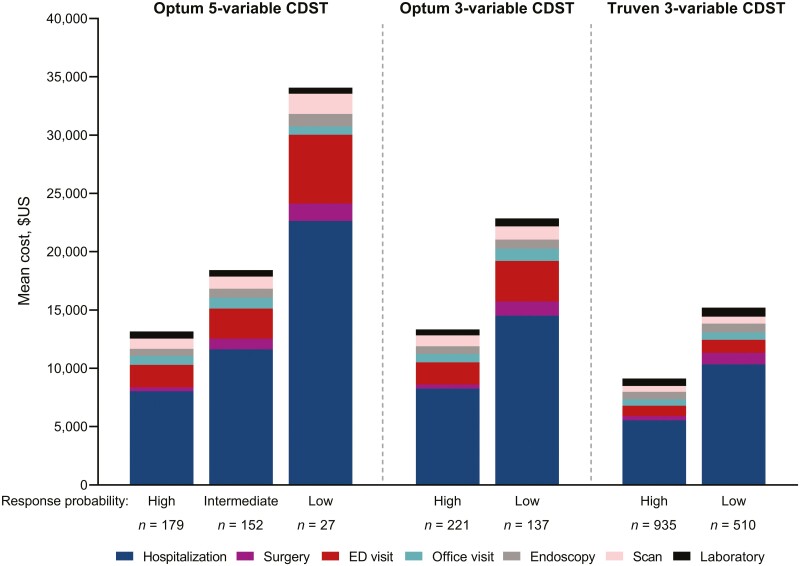
Annualized disease-related health expenditures in patients with CD treated with vedolizumab (Optum and Truven data sets). Abbreviations: CD, Crohn’s disease; CDST, clinical decision support tool; ED, emergency department.

A significant association was observed between mean healthcare costs and baseline probability of response for vedolizumab-treated CD patients but not anti-TNF-treated CD patients (Table 2, Figure 3, Supplementary Table S13). Among vedolizumab-treated CD patients, total CD-related healthcare costs were significantly different (*P* = .017) between the three 5-variable CDST probability of response groups, with a 2- to 3-fold difference between high (mean [SD], $12 943.0 [$37 738.7]), intermediate ($17 944.2 [$26 623.0]) and low ($32 391.2 [$49 849.4]) probabilities of response groups, and the lowest costs observed in the high response group. This relationship was also observed using the modified 3-variable CDST (mean, $13 067.3 vs $22 230.5, *P* = .0156). Conversely, among anti-TNF-treated CD patients, there was no significant association between mean healthcare costs and baseline probability of response using either the full 5-variable or modified 3-variable CDST (Supplementary Figure S2).

**Table 2.** Annualized CD-related health expenditures of patients with CD treated with vedolizumab (Optum and Truven data sets).

Cost ($) of CD-related events, mean (SD)	Optum data set							Truven data set		
	5-variable CDST				3-variable CDST			3-variable CDST		
	Probability of response			*P* value^a^	Probability of response		*P* value^a^	Probability of response		*P* value^a^
	High (*n* = 179)	Int (*n* = 152)	Low (*n* = 27)		High (*n* = 221)	Low (*n* = 137)		High (*n* = 935)	Low (*n* = 510)	
Hospitalization	8018.2 (28 677.8)	11 642.1 (23 376.7)	22 625.8 (42 506.3)	.0350^*^	8265.7 (26 690.6)	14 518.5 (29 841.9)	.0403^*^	5532.3 (19 277.7)	10 341.8 (29 943.2)	.0002^*^
Surgery	354.5 (1605.3)	925.5 (2355.6)	1507.0 (2315.8)	.0035^*^	352.8 (1514.2)	1217.9 (2593.7)	<.0001^*^	378.9 (1281.0)	976.3 (2757.8)	<.0001^*^
ED visit	1919.1 (11 042.4)	2579.0 (7328.8)	5902.5 (15 235.1)	.1585	1898.9 (10 157.6)	3468.9 (9895.1)	.1520	871.2 (4399.6)	1126.7 (4355.2)	.2899
All events^b^	12 943.0 (37 738.7)	17 944.2 (26 623.0)	32 391.2 (49 849.4)	.0170^*^	13 067.3 (34 676.2)	22 230.5 (34 659.6)	.0156^*^	8842.2 (21 161.7)	14 591.8 (31 776.2)	<.0001^*^

Abbreviations: CD, Crohn’s disease; CDST, clinical decision support tool; ED, emergency department; Int, intermediate.

^a^ *P* values are from the χ^2^-test for categorical variables and 2-sample *t*-test for continuous variables.

^b^Includes CD-related hospitalization, surgery, ED visits, office visits, endoscopy, scans, and laboratory tests.

^*^ *P* values are statistically significant.

#### Truven data set

Mean healthcare costs followed the same pattern for vedolizumab-treated CD patients in the Truven database, although mean costs were lower compared with the Optum data set (Table 2; Figure 3). Patients with a high baseline probability of response had significantly lower healthcare expenditure costs during the 12 months after treatment initiation (mean, $8842.2 vs $14 591.8, *P* < .0001), which was driven by the lower costs incurred for CD-related hospitalization and surgery.

## Discussion

With the increasing use of biologic agents for the treatment of IBD over the last decade,^17,18^ clinical prediction models that estimate the probability of a desired outcome can help guide physicians in treatment decisions.^19,20^ The full 5-variable vedolizumab CDST, previously developed using data from the phase 3 GEMINI 2 trial and validated using data from the real-world VICTORY consortium, was observed to identify CD patients most likely to respond to 26 weeks of vedolizumab therapy.^15^ Given that CD patients may require surgery during the course of disease, initial treatment selection, and identification of those patients who are more likely to respond to a specific therapy is important^3^ and may eventually lead to reduced overall costs of care.

In this study, we extended the utility of this CDST to apply clinical trial findings to predict real-world outcomes and inform decisions on patient care. Real-world data were assessed to determine how the full 5-variable CDST can be used in clinical practice and in the prediction of HRU when implemented at the population level. We tested the full 5-variable model versus a modified 3-variable model that does not require laboratory values to help to address the inherent heterogeneity of, and limitations on, data availability associated with real-world data. Both tools showed that patients with a high probability of vedolizumab response had favorable outcomes and less annualized healthcare expenditures. The full 5-variable CDST and the modified 3-variable CDST showed comparable outcomes for patients treated with vedolizumab using the Optum data set, and both tools were better at predicting outcomes for patients treated with vedolizumab than those treated with an anti-TNF. The high-probability vedolizumab response group had significantly lower total healthcare expenditure costs than the low-probability group using both the full 5-variable and the modified 3-variable CDST, while consistent significant differences in healthcare costs were not observed for anti-TNF-treated patients when stratified by the CDST.

This study has some limitations. For example, although the current study has demonstrated the effectiveness of the CDST tool for identifying patients treated with vedolizumab who are at higher risk for HRU, as well as demonstrating relative lack of effectiveness for anti-TNF-treated patients, it does not assess the effectiveness of the tool for other biologics, including ustekinumab. Nonetheless, another CDST designed specifically for ustekinumab has been developed and assessed in patients with CD.^21^ Although there are some common features between the vedolizumab CDST and the ustiknumab CDST, they are not identical and have sufficient differences in variables to mean that each is likely to be selective for the drug for which it was designed.^22^ Another limitation of the retrospective study was that the data did not allow comparison of treatment patterns within groups. We are unable, for example, to determine if anti-TNF-treated patients with low probability of response might have different outcomes and costs depending on the precise treatment given (eg, dose optimized vs standard drug dosing). Consequently, while this tool can be used to identify at-risk patients and direct clinicians to appropriate treatment strategies, including treat-to-target monitoring, how well the tool predicts the actual outcomes for individual patients may vary depending on the treatment provided. A final limitation is the fact that source administrative data may lack clinical detail, resulting in possible misclassification of patients. Those with mild CD may not have sought medical attention or utilized healthcare resources and hence may have not been identified as part of the data set. However, patients with moderately to severely active CD are expected to have greater burden of disease and hence may account for the majority of the cost of care. Formal cost-effectiveness analyses are recommended to guide clinical decisions further on the relative effectiveness of vedolizumab and other therapeutic options for CD with regard to HRU.

## Conclusions

This study confirms that our vedolizumab CDST, which was originally developed from clinical trial data sets, can be used to analyze real-world data to predict patient HRU. Both the full 5-variable and the modified 3-variable CDSTs can be used to identify CD patients treated with vedolizumab who are at higher risk for HRU, especially for hospitalization and surgery; our new analysis shows that the tool is effective for vedolizumab-treated patients but cannot be reliably used for patients receiving anti-TNF treatments. These results support integration of the 5- and 3-variable vedolizumab CDSTs into IBD population health monitoring algorithms using real-world data.

## Supplementary Material

otac048_suppl_Supplementary_Material

otac048_suppl_Supplementary_Figure_S1

otac048_suppl_Supplementary_Figure_S2

## Data Availability

The data that support the findings of this study are available on request from the corresponding author. The data are not publicly available due to privacy or ethical restrictions.
