# Supplementary material for: Probability of Response as Defined by a Clinical Decision Support Tool Is Associated With Lower Healthcare Resource Utilization in Vedolizumab-Treated Patients With Crohn’s Disease
Source: Crohns Colitis 360. 2022 Dec 3;4(4):otac048. doi: 10.1093/crocol/otac048 (PMC9802432; doi:10.1093/crocol/otac048)
Supplement: otac048_suppl_Supplementary_Material [file otac048_suppl_supplementary_material.docx]

**Probability of Response as Defined by a Clinical Decision Support Tool is Associated With Lower Healthcare Resource Utilization in Vedolizumab-treated Patients With Crohn’s Disease**

Supplementary information

**Supplementary Table 1.** Codes used for identification of patients with Crohn’s disease treated with vedolizumab

**Supplementary Table 2.** Diagnosis codes used for identification of patients with Crohn’s disease or ulcerative colitis

**Supplementary Table 3.** Anti-tumor necrosis factor therapies

**Supplementary Table 4.** Corticosteroid medications

**Supplementary Table 5.** Immunomodulatory agents and 5-aminosalicylic acids

**Supplementary Table 6.** Diagnosis codes used to identify patients with fistula disease

**Supplementary Table 7.** 23-hour observation CPT codes

**Supplementary Table 8.** Surgery CPT codes

**Supplementary Table 9.** Calculation of the ≤3-point cut-off for defining high and low probability of response using the modified CDST (no laboratory values)

**Supplementary Table 10.** Endoscopy CPT codes

**Supplementary Table 11.** Scan CPT codes

**Supplementary Table 12.** Baseline demographics and characteristics of Crohn’s disease patients treated with an anti-TNF agent (Optum data set)

**Supplementary Table 13.** Annualized Crohn’s disease-related health expenditures in Crohn’s disease patients treated with an anti-TNF agent (Optum data set)

**Supplementary Figure 1.** Healthcare resource utilization in Crohn’s disease patients treated with an anti-TNF agent (Optum data set)

**Supplementary Figure 2.** Annualized Crohn’s disease-related health expenditures in Crohn’s disease patients treated with an anti-TNF agent (Optum data set)

**Supplementary Table 1.** Codes used for identification of patients with Crohn’s disease treated with vedolizumab

| Active ingredient | Brand name | Approval date | NDC | HCPCS code | Code description | Service unit | Pay |
| --- | --- | --- | --- | --- | --- | --- | --- |
| Vedolizumab | Entyvio^®^ | May-2014 | 64764-0300-20 | C9026 | Injection, vedolizumab 1 mg | Any amount | Any amount |
|  |  |  |  | J3380 | Injection, vedolizumab 1 mg | Any amount | Any amount |

Abbreviations: HCPCS, healthcare common procedure coding system; NDC, national drug code.

**Supplementary Table 2.** Diagnosis codes used for identification of patients with Crohn’s disease or ulcerative colitis

| ICD-9 diagnosis codes | | ICD-10 diagnosis codes | |
| --- | --- | --- | --- |
| Code | **Description** | **Code** | **Description** |
| 555.xx | Crohn’s disease | K50.xx | Crohn’s disease |
| 556.xx | Ulcerative colitis | K51.xx | Ulcerative colitis |

Abbreviation: ICD, international classification of diseases.

**Supplementary Table 3.** Anti-tumor necrosis factor therapies

| Active ingredient | Brand name | Approval date | NDC | HCPCS code |
| --- | --- | --- | --- | --- |
| Adalimumab | Humira | Jan-2003 | 00074-0067-02  00074-0124-01  00074-0124-03  00074-0124-73  00074-0124-74  00074-0243-02  00074-0243-71  00074-0554-01  00074-0554-02  00074-0554-04  00074-0554-06  00074-0554-71  00074-0554-73  00074-0554-74  00074-0616-02  00074-0616-71  00074-0817-02  00074-1539-03  00074-2540-01  00074-2540-03  00074-3797-01  00074-3799-02  00074-3799-03  00074-3799-06  00074-3799-71  00074-4339-01  00074-4339-02  00074-4339-06  00074-4339-07  00074-4339-71  00074-4339-73  00074-4339-74  00074-6347-02  00074-9374-02  00074-9374-71  00074-3797-02  00074-3799-01 | J0135 |
| Certolizumab pegol | Cimzia | May-2009 | 50474-0700-61  50474-0700-62  50474-0710-79  50474-0710-80  50474-0710-81 | C9249, J0717, J0718 |
| Golimumab | Simponi | Apr-2009 | 57894-0070-01  57894-0070-02  57894-0070-89  57894-0070-90  57894-0071-01  57894-0071-02  57894-0071-89  57894-0071-90  57894-0350-01  57894-0350-89 | J1602 |
| Infliximab | Remicade | Aug-1998 | 57894-0030-01 | J1745  Biosimilar: Q5102 |

Abbreviations: HCPCS, healthcare common procedure coding system; NDC, national drug code.

**Supplementary Table 4.** Corticosteroid medications

| Medication class | THERDTL code | HCPCS code |
| --- | --- | --- |
| Betamethasone | 6804010015 | J0704 |
| Budesonide | 6804010019 |  |
| Cortisone | 6804010020 | J0810 |
| Dexamethasone | 6804010030 | J1094, J1095, J1100, J8540, S0173 |
| Hydrocortisone | 6804010050 | J1700, J1710, J1720 |
| Methylprednisolone | 6804010060 | J1020, J1030, J1040, J2920, J2930, J7509 |
| Prednisolone | 6804010070 | J1690, J2640, J2650 |
| Prednisone | 6804010075 | J7506, J7510 |
| Triamcinolone | 6804010085 | J3301, J3302, J3303 |

Abbreviations: HCPCS, healthcare common procedure coding system; THERDTL, therapeutic detail.

**Supplementary Table 5.** Immunomodulatory agents and 5-aminosalicylic acids

| Medication class | THERDTL code | HCPCS code |
| --- | --- | --- |
| Cyclosporine | 7001010030 | C9438, J7502, J7503, J7515, J7516, K0122, K0121, K0418 |
| Leflunomide | 7001010057 |  |
| Lenalidomide | 9220000045 |  |
| Methotrexate | 1005010195 | J8610, J9250, J9260 |
| Mycophenolate mofetil | 7001010063 | J7517, K0412 |
| Sirolimus | 7001010064 | J7520 |
| Tacrolimus | 7001010065 | C9006, J7508, J7507, J7525 |
| Thalidomide | 9220000065 |  |
| Thiopurines  6-mercaptopurine  Azathioprine  Thioguanine | 1005010190  7001010020  1005010315 | S0108  J7500, J7501 |
| 5-aminosalicylic acid  Balsalazide  Olsalazine  Sulfasalazine  Mesalamine | 5640010004  5640010050  824010040  5640010030 |  |

Abbreviations: HCPCS, healthcare common procedure coding system; THERDTL, therapeutic detail.

**Supplementary Table 6.** Diagnosis codes used for identification of patients with fistula disease

| ICD-9 diagnosis codes | | | |
| --- | --- | --- | --- |
| ICD-9 CM codes | **Description** | **ICD-9 PCS codes** | **Description** |
| 565.1 | Anal fistula | 4284 | Repair of esophageal fistula, not elsewhere classified |
| 569.81 | Fistula of intestine, excluding rectum and anus | 4463 | Closure of other gastric fistula |
| 5374 | Fistula of stomach or duodenum | 4672 | Closure of fistula of duodenum |
| 5961 | Intestinovesical fistula | 4674 | Closure of fistula of small intestine, except duodenum |
| 6191 | Digestive-genital tract fistula, female | 4676 | Closure of fistula of large intestine |
| 9986 | Persistent postoperative fistula | 4792 | Closure of appendiceal fistula |
|  |  | 4873 | Closure of other rectal fistula |
|  |  | 4893 | Repair of perirectal fistula |
|  |  | 4973 | Closure of anal fistula |
|  |  | 5783 | Repair of fistula involving bladder and intestine |
|  |  | 7072 | Repair of colovaginal fistula |
|  |  | 7073 | Repair of rectovaginal fistula |
|  |  | 7074 | Repair of other vaginoenteric fistula |
|  |  | 7172 | Repair of fistula of vulva or perineum |
|  |  | 49.11 | Anal fistulotomy |
|  |  | 49.12 | Anal fistulectomy |
| ICD-10 diagnosis codes | | | |
| Crohn’s disease-specific  ICD-10 CM codes | **Description** | **Nonspecific**  **ICD-10 CM codes** | **Description** |
| K50.013 | Crohn's disease of small intestine with fistula | K316 | Fistula of stomach and duodenum |
| K50.113 | Crohn's disease of large intestine with fistula | K383 | Fistula of appendix |
| K50.813 | Crohn's disease of both small and large intestine with fistula | K603 | Anal fistula |
| K50.913 | Crohn's disease, unspecified, with fistula | K604 | Rectal fistula |
|  |  | K605 | Anorectal fistula |
|  |  | K632 | Fistula of intestine |
|  |  | N321 | Vesicointestinal fistula |
|  |  | N822 | Fistula of vagina to small intestine |
|  |  | N823 | Fistula of vagina to large intestine |
|  |  | N824 | Other female intestinal-genital tract fistulae |
| CPT codes | | | |
| Code | **Description** | **Code** | **Description** |
| 43305 | Repair esophagus and fistula | 46275 | Remove anal fist inter |
| 43312 | Repair esophagus and fistula | 46280 | Remove anal fist complex |
| 43880 | Repair stomach-bowel fistula | 46285 | Remove anal fist 2 stage |
| 44640 | Repair bowel-skin fistula | 46288 | Repair anal fistula |
| 44650 | Repair bowel fistula | 46288 | Repair anal fistula |
| 44660 | Repair bowel-bladder fistula | 46706 | Repr of anal fistula w/glue |
| 44661 | Repair bowel-bladder fistula | 46706 | Repr of anal fistula w/glue |
| 45800 | Repair rect/bladder fistula | 46707 | Repair anorectal fist w/plug |
| 45805 | Repair fistula w/colostomy | 46715 | Rep perf anoper fistu |
| 45820 | Repair rectourethral fistula | 46716 | Rep perf anoper/vestib fistu |
| 45825 | Repair fistula w/colostomy | 50930 | Closure ureter/bowel fistula |
| 46020 | Placement of seton | 57300 | Repair rectum-vagina fistula |
| 46258 | Remove in/ex hem grp w/fistu | 57305 | Repair rectum-vagina fistula |
| 46262 | Remove in/ex hem grps w/fist | 57307 | Fistula repair & colostomy |
| 46270 | Remove anal fist subq | 57308 | Fistula repair transperine |

Abbreviations: CM, clinical modification; CPT, current procedural terminology; ICD, international classification of diseases; PCS, procedure coding system.

**Supplementary Table 7.** 23-hour observation CPT codes

| CPT code | Description |
| --- | --- |
| 99218 | Initial observation care |
| 99219 | Initial observation care |
| 99220 | Initial observation care |
| 99224 | Subsequent observation care |
| 99225 | Subsequent observation care |
| 99226 | Subsequent observation care |

Abbreviation: CPT, current procedural terminology.

**Supplementary Table 8.** Surgery CPT codes

| CPT code | Description |
| --- | --- |
| 44139, 44140, 44141, 44143, 44144, 44145, 44146, 44147, 44160, 44204, 44205, 44206, 44207, 44208, 44213 | Partial colectomy |
| 44150, 44151, 44152, 44153, 44155, 44156, 44157, 44158, 44210, 44211, 44212 | Total colectomy |
| 44015, 44130, 44186, 44187, 44188, 44227, 44300, 44310, 44312, 44314, 44316, 44320, 44322, 44340, 44345, 44346, 45395, 45397, 44620, 44625, 44626, 45136 | Ostomy (Colostomy or Ileostomy) |
| 44640, 44650, 44660, 44661, 45800, 45805, 45820, 45825, 46020, 46030, 46258, 46262, 46270, 46275, 46280, 46285, 46288, 46706, 46710, 46712, 46715, 46716, 46740, 46742, 57300, 57305, 57307, 57308. Also include codes in Appendix G under “ICD-9 PCS Codes” and “CPT Codes”. | Fistula (only for Crohn’s disease patients) |
| 10060, 10061, 10140, 10160, 10180, 45000, 45005, 45020, 46040, 46045, 46050, 46060, 49020, 49040, 49060, 49062 | Abscess |
| 44615, 45150, 46700, 46705, 4891(ICD-9 PCS Code) | Stricture |
| 44602, 44603, 44604, 44605, 44700, 44701, 45500, 45505, 45520, 45540, 45541, 45550, 45560, 45562, 45563, 45900, 45905, 45910, 45915 | Anal/rectal repair |
| 44120, 44121, 44125, 44126, 44127, 44128, 44202, 44203 | Enterectomy |
| 45110, 45111, 45112, 45113, 45114, 45116, 45119, 45120, 45121, 45123, 45126 | Proctectomy |
| 44005, 49000 | Other (exploratory laparotomy and lysis of adhesions) |

Abbreviation: CPT, current procedural terminology.

**Supplementary Table 9.** Calculation of the ≤3-point cut-off point for defining high and low probability of response using the modified CDST (no laboratory values)

| 5-variable CDST | | 3-variable CDST^a^ | | |
| --- | --- | --- | --- | --- |
|  |  | **High** | **Low** | **Total** |
| High | n | 158 | 16 | 174 |
|  |  | 39.50 | 4.00 | 43.50 |
|  |  | **90.80** | 9.20 |  |
|  |  | 77.83 | 8.12 |  |
| Low | n | 45 | 181 | 226 |
|  |  | 11.25 | 45.25 | 56.50 |
|  |  | 19.91 | **80.09** |  |
|  |  | 22.17 | 91.88 |  |
| Total | n | 203 | 197 | 400 |
|  |  | 50.75 | 49.25 | 100.00 |

Abbreviation: CDST, clinical decision support tool.

^a^High and low response was defined using a cut-off point at 3 points (out of a possible 7 points).

**Supplementary Table 10.** Endoscopy CPT codes

| CPT code | Description |
| --- | --- |
| 44360 | Small bowel endoscopy |
| 44361 | Small bowel endoscopy/biopsy |
| 44376 | Small bowel endoscopy |
| 44377 | Small bowel endoscopy/biopsy |
| 44380 | Small bowel endoscopy br/wa |
| 44381 | Small bowel endoscopy br/wa |
| 44382 | Small bowel endoscopy |
| 44388 | Colonoscopy thru stoma spx |
| 44389 | Colonoscopy with biopsy |
| 44390 | Colonoscopy for foreign body |
| 44391 | Colonoscopy for bleeding |
| 44392 | Colonoscopy & polypectomy |
| 44393 | Colonoscopy with ablation |
| 44394 | Colonoscopy w/snare |
| 44397 | Colonoscopy with stent |
| 45300 | Proctosigmoidoscopy dx |
| 45303 | Proctosigmoidoscopy dilate |
| 45305 | Proctosigmoidoscopy w/bx |
| 45307 | Proctosigmoidoscopy fb |
| 45308 | Proctosigmoidoscopy removal |
| 45309 | Proctosigmoidoscopy removal |
| 45315 | Proctosigmoidoscopy removal |
| 45317 | Proctosigmoidoscopy bleed |
| 45320 | Proctosigmoidoscopy ablate |
| 45321 | Proctosigmoidoscopy volvul |
| 45327 | Proctosigmoidoscopy w/stent |
| 45330 | Diagnostic sigmoidoscopy |
| 45331 | Sigmoidoscopy and biopsy |
| 45332 | Sigmoidoscopy w/fb removal |
| 45333 | Sigmoidoscopy & polypectomy |
| 45334 | Sigmoidoscopy for bleeding |
| 45335 | Sigmoidoscopy w/submuc inj |
| 45337 | Sigmoidoscopy & decompress |
| 45338 | Sigmoidoscopy w/tumr remove |
| 45339 | Sigmoidoscopy w/ ablation |
| 45340 | Sig w/tndsc balloon dilation |
| 45341 | Sigmoidoscopy w/ultrasound |
| 45342 | Sigmoidoscopy w/aspiration |
| 45345 | Sigmoidoscopy w/stent |
| 45355 | Colonoscopy via colotomy |
| 45378 | Diagnostic colonoscopy |
| 45379 | Colonoscopy w/fb removal |
| 45380 | Colonoscopy and biopsy |
| 45381 | Colonoscopy submucous njx |
| 45382 | Colonoscopy w/control bleed |
| 45383 | Colonoscopy w/ ablation |
| 45384 | Colonoscopy w/lesion removal |
| 45385 | Colonoscopy w/lesion removal |
| 45386 | Colonoscopy w/balloon dilat |
| 45387 | Colonoscopy w/stent plcmt |
| 45388 | Colonoscopy w/ablation |
| 45389 | Colonoscopy w/stent plcmt |
| 45390 | Colonoscopy w/resection |
| 45392 | Colonoscopy w/endoscopic fnb |
| 45393 | Colonoscopy w/decompression |
| 45398 | Colonoscopy w/band ligation |
| 45399 | Unlisted procedure colon |

Abbreviation: CPT, current procedural terminology.

**Supplementary Table 11.** Scan CPT codes

| CPT code | Description |
| --- | --- |
| 74000 | X-ray exam of abdomen |
| 74020 | X-ray exam of abdomen |
| 74150 | CT abdomen w/o dye |
| 74160 | CT abdomen w/dye |
| 74170 | CT abdomen w/o & w/dye |
| 74174 | CT angio abd&pelv w/o & w/dye |
| 74175 | CT angio abdom w/o & w/dye |
| 74176 | CT abd & pelvis w/o contrast |
| 74177 | CT abd & pelv w/contrast |
| 74178 | CT abd & pelv 1/> regns |
| 74181 | MRI abdomen w/o dye |
| 74183 | MRI abdomen w/o & w/dye |
| 74185 | MRI angio abdom w or w/o dye |
| 76700 | Us exam abdom complete |
| 76705 | Echo exam of abdomen |

Abbreviations: CPT, current procedural terminology; CT, computed tomography; MRI, magnetic resonance imaging.

**Supplementary Table 12.** Baseline demographics and characteristics of patients with CD treated with an anti-TNF agent (Optum data set)

|  | Optum data set | | | | | | |
| --- | --- | --- | --- | --- | --- | --- | --- |
|  | **5-variable CDST** | | | | **3-variable CDST** | | |
|  | **Probability of response** | | |  | **Probability of response** | |  |
|  | **High**  **(*n* = 650)** | **Intermediate**  **(*n* = 158)** | **Low**  **(*n* = 6)** | ***P* value** | **High**  **(*n* = 726)** | **Low**  **(*n* = 88)** | ***P* value** |
| Female, *n* (%) | 358 (55.1) | 87 (55.1) | 2 (33.3) | 0.566 | 409 (56.3) | 38 (43.2) | 0.019* |
| Age in years, mean (SD) | 41.8 (15.3) | 44.2 (17.0) | 44.3 (16.9) | 0.203 | 42.3 (15.7) | 42.4 (15.6) | 0.957 |
| Disease duration in years, mean (SD) | 2.7 (3.6) | 2.8 (3.7) | 1.1 (1.0) | 0.543 | 2.6 (3.5) | 3.6 (4.0) | 0.019* |
| CD-related hospitalization,^a^ *n* (%) | 125 (19.2) | 65 (41.1) | 2 (33.3) | <0.001* | 158 (21.8) | 34 (38.6) | <0.001* |
| CD-related surgery,^a^ *n* (%) | 43 (6.6) | 49 (31.0) | 2 (33.3) | <0.001* | 39 (5.4) | 55 (62.5) | <0.001* |
| Fistula,^a^ *n* (%) | 71 (10.9) | 68 (43.0) | 5 (83.3) | <0.001* | 72 (9.9) | 72 (81.8) | <0.001* |
| Stricture,^a^ *n* (%) | 1 (0.2) | 0 (0.0) | 0 (0.0) | 0.881 | 1 (0.1) | 0 (0.0) | 0.728 |
| History of fistulizing disease, *n* (%) | 83 (12.8) | 80 (50.6) | 5 (83.3) | <0.001* | 80 (11.0) | 88 (100.0) | <0.001* |
| IMM- or anti-TNF-naïve, *n* (%) | 429 (66.0) | 98 (62.0) | 6 (100.0) | 0.130 | 478 (65.8) | 55 (62.5) | 0.534 |
| Concomitant IMM treatment, *n* (%) | 161 (24.8) | 45 (28.5) | 2 (33.3) | 0.573 | 186 (25.6) | 22 (25.0) | 0.900 |
| Concomitant CS treatment, *n* (%) | 301 (46.3) | 93 (58.9) | 4 (66.7) | 0.012* | 357 (49.2) | 41 (46.6) | 0.647 |

Abbreviations: CD, Crohn’s disease; CDST, clinical decision support tool; CS, corticosteroid; IMM, immunomodulator; SD, standard deviation; TNF, tumor necrosis factor.

**P* values are statistically significant.

^a^During the year before vedolizumab initiation.

**Supplementary Table 13.** Annualized CD-related health expenditures in patients with CD treated with an anti-TNF agent (Optum data set)

| Cost ($) of  CD-related events, mean (SD) | 5-variable CDST^c^ | | | 3-variable CDST | | |
| --- | --- | --- | --- | --- | --- | --- |
|  | **Probability of response** | |  | **Probability of response** | |  |
|  | **High**  **(N = 650)** | **Intermediate**  **(N = 158)** | ***P* value^a^** | **High**  **(N = 726)** | **Low**  **(N = 88)** | ***P* value^a^** |
| Hospitalization | 5203.7 (16 880.9) | 6339.7 (23 615.7) | 0.4863 | 5593.6 (18 853.0) | 4487.6 (13 872.5) | 0.5942 |
| Surgery | 323.2 (1334.7) | 1216.7 (6150.7) | 0.0007* | 442.5 (2964.6) | 1154.6 (3534.8) | 0.0377* |
| ED visit | 1517.3 (5791.6) | 1832.4 (6405.3) | 0.5484 | 1681.5 (6190.5) | 867.2 (2449.7) | 0.2221 |
| All events^b^ | 8602.2 (20 127.5) | 11 053.6 (27 439.9) | 0.2041 | 9350.4 (22 468.1) | 9908.9 (26 237.6) | 0.8290 |

Abbreviations: CD, Crohn’s disease; CDST, clinical decision support tool; ED, emergency department; SD, standard deviation; TNF, tumor necrosis factor.**P* values are statistically significant.

^a^*P* values are from the χ^2^-test for categorical variables and two-sample *t*-test for continuous variables.

^b^Includes CD-related hospitalization, surgery, ED visits, office visits, endoscopy, scans, and laboratory tests.

^c^Low-probability responders were not included due to the small number of anti-TNF-treated patients classified with low probability of response using the full 5‑variable CDST (N = 6).

**Supplementary Figure 1.** Healthcare resource utilization in CD patients treated with an anti-TNF agent (Optum data set)^a^

**

**

Abbreviations: CD, Crohn’s disease; CDST, clinical decision support tool; ED, emergency department; TNF, tumor necrosis factor.

*P* values are from χ2-tests; *P* < 0.05 is statistically significant.

^a^Low-probability responders were not included due to the small number of anti-TNF–treated patients classified with low probability of response using the full 5‑variable CDST (N = 6).

**Supplementary Figure 2.** Annualized CD-related health expenditures in patients treated with an anti-TNF agent (Optum data set)^a^

**
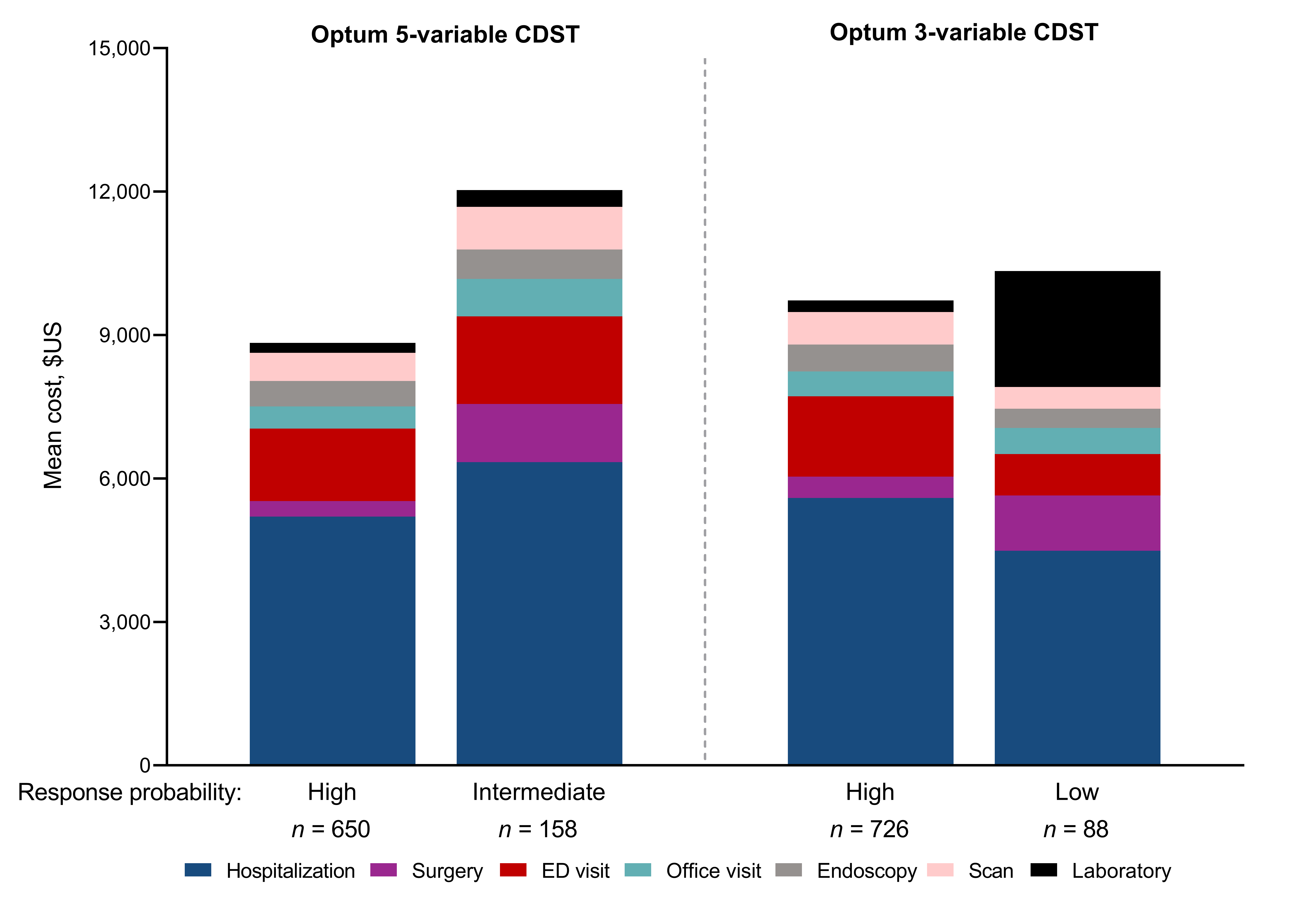
**

Abbreviations: CD, Crohn’s disease; CDST, clinical decision support tool; ED, emergency department; TNF, tumor necrosis factor.

^a^Low-probability responders were not included due to the small number of anti-TNF–treated patients classified with low probability of response using the full 5‑variable CDST (N = 6).
