# Supplementary figures and images for: Probability of Response as Defined by a Clinical Decision Support Tool Is Associated With Lower Healthcare Resource Utilization in Vedolizumab-Treated Patients With Crohn’s Disease
Source: Crohns Colitis 360. 2022 Dec 3;4(4):otac048. doi: 10.1093/crocol/otac048 (PMC9802432; doi:10.1093/crocol/otac048)

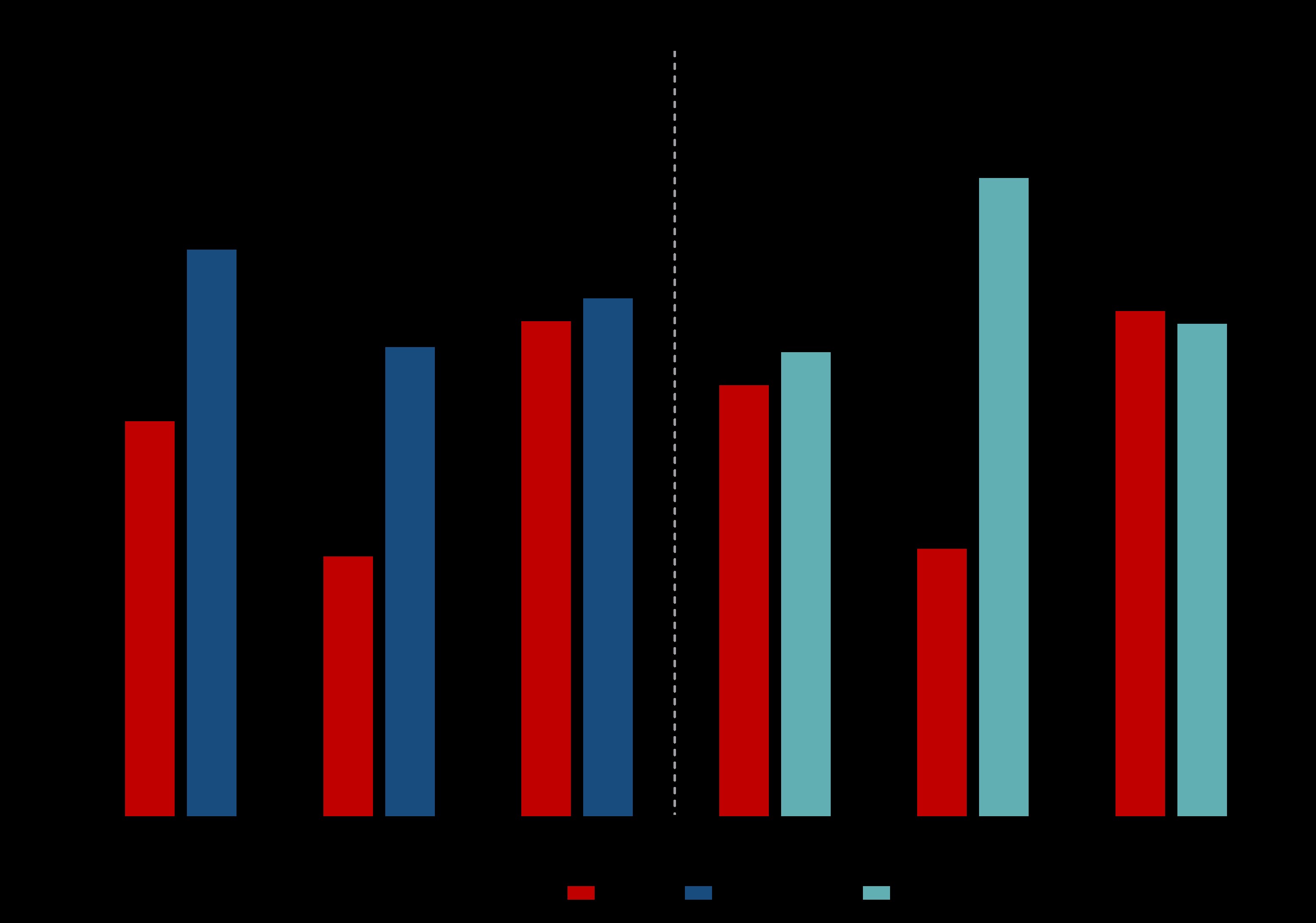

Supplement: otac048_suppl_Supplementary_Figure_S1 [file otac048_suppl_supplementary_figure_s1.jpeg]

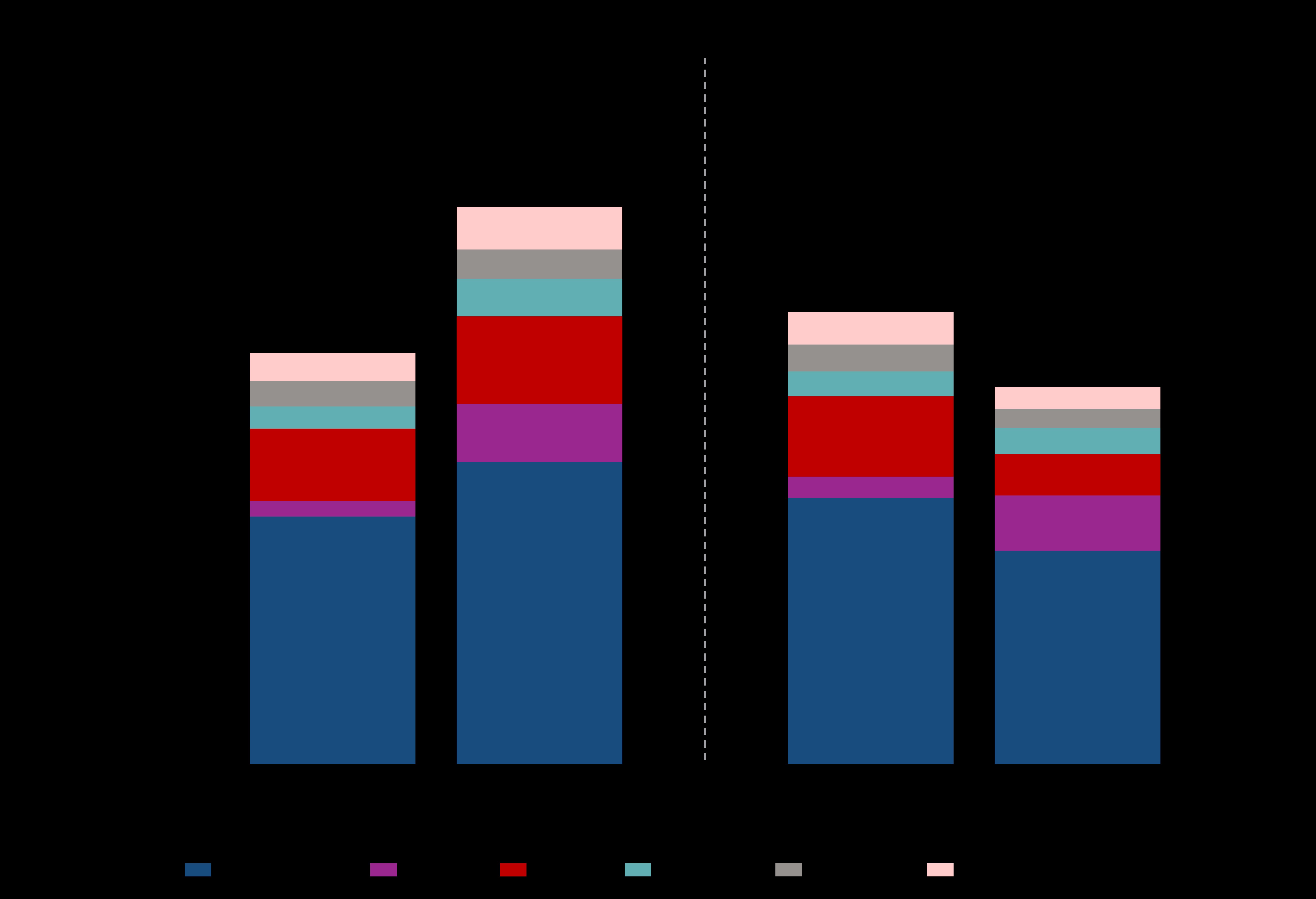

Supplement: otac048_suppl_Supplementary_Figure_S2 [file otac048_suppl_supplementary_figure_s2.jpeg]
